# Role of TGFβ-activated cancer-associated fibroblasts in the resistance to checkpoint blockade immunotherapy

**DOI:** 10.3389/fonc.2025.1602452

**Published:** 2025-05-21

**Authors:** Weibin Hou

**Affiliations:** Department of Urology, the Third Xiangya Hospital, Central South University, Changsha, China

**Keywords:** cancer associated fibroblasts (CAFs), immune checkpoint blockers (ICBs), transforming growth factor beta (TGFβ), immunotherapy resistance, T cell exclusion, myofibroblast

## Abstract

Immune checkpoint blockers (ICBs) have revolutionized cancer treatment by enabling durable responses. However, most patients showed resistance and limited efficacy. Elucidating mechanisms of resistance is imperative to extend the clinical utility of ICBs. Emerging evidence highlights cancer-associated fibroblasts (CAFs), particularly TGFβ-activated myofibroblastic CAFs, as key orchestrators of immunosuppressive TMEs and ICBs resistance. These CAFs drive T-cell exclusion preventing intratumoral T cells from engaging cancer cells. This review explores the role of TGFβ signaling in CAFs in driving immune evasion and therapy resistance. While targeting TGFβ or CAFs directly has shown limited inconsistent results, downstream molecules in TGFβ-activated CAFs, including induced TGFβ (βig-h3), endocytic receptor 180 (Endo180), leucine-rich repeat containing 15 (LRRC15), and NADPH oxidase 4 (NOX4), emerge as promising therapeutic targets to counteract T-cell exclusion and restore immunotherapy sensitivity. Advancing research on CAF heterogeneity and pro-tumorigenic subsets may uncover innovative strategies to expand immunotherapy benefits.

## 1 Introduction

Immune checkpoint blockers (ICBs), especially PD1/PDL1 inhibitors, have revolutionized cancer treatment due to their potential for durable responses and long-term disease control. However, only a subset of patients benefit from ICBs, with most being resistant (1). The efficacy of ICBs depends on pre-existing T-cell infiltration into tumors. However, high T-cell density alone does not guarantee a response. The spatial distribution of T cells is also critical, with physical interaction between T cells and cancer cells required for ICB efficacy (1).

Solid tumors are currently stratified into the following three distinct immune phenotypes based on T-cell spatial distribution (2): 1) inflamed/hot tumors, characterized by abundant intratumoral T-cell infiltration, yet frequently resistant to ICIs due to upregulation of alternative checkpoints (TIM3, LAG3, TIGIT) and non-PD-1/CTLA4-mediated inhibitory pathways (3, 4); 2) desert/cold tumors, defined by minimal T-cell presence, primarily attributed to defective antigen presentation or failed T-cell priming necessitating combinatorial strategies with immunogenic priming agents (e.g., neoantigen vaccines, oncolytic viruses) (5); and 3) immune-excluded tumors, distinguished by CD8+ T-cell sequestration within tumor stroma, forming a “peripheral immune shield”—host-derived antitumor T cells are physically barred from infiltrating cancer nests resulting in poor prognosis and primary resistance to ICIs (6).

**Figure 1:**
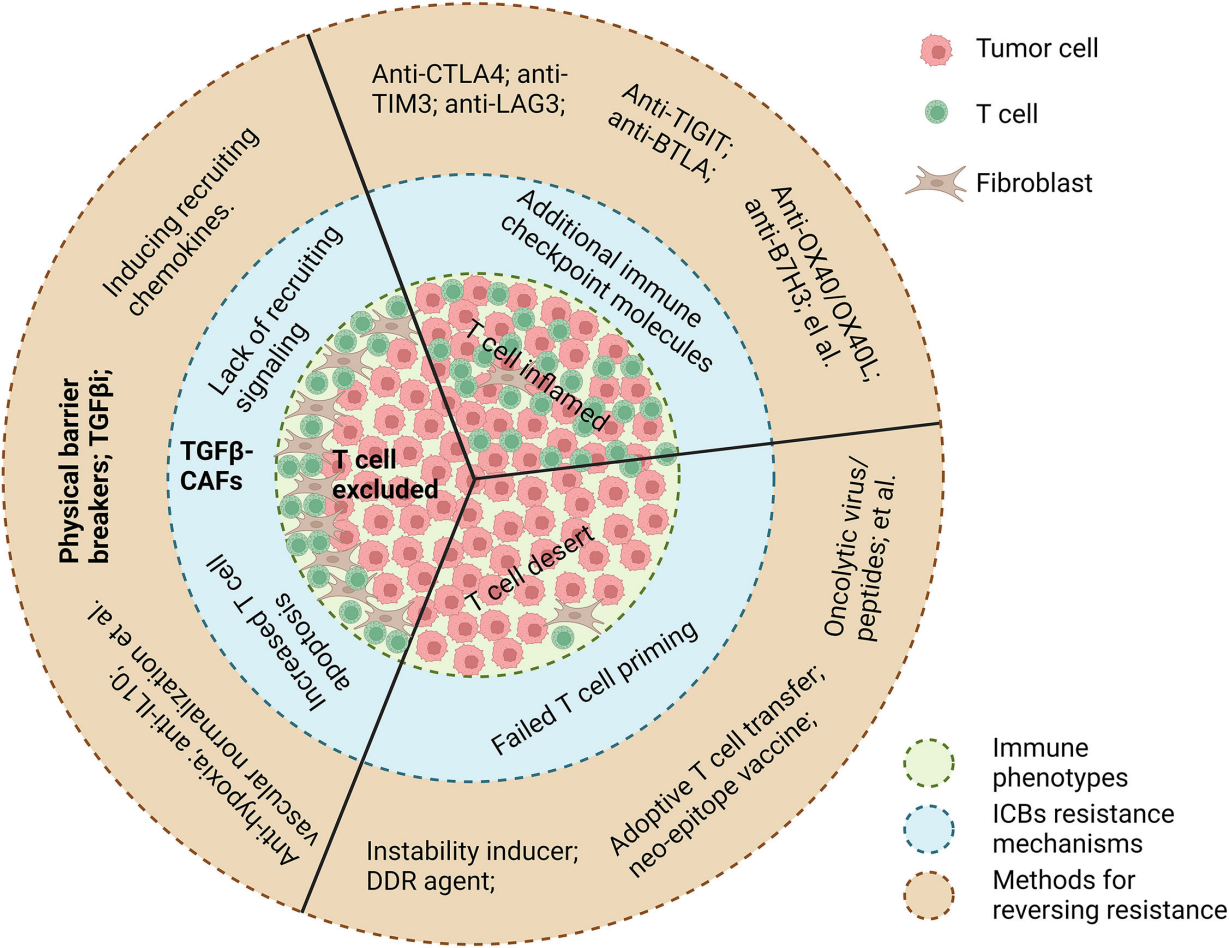
A summary of current knowledge on ICB resistance mechanisms underlying three immune phenotypes, respectively, and their corresponding methods for reversing resistance. Image created with Biorender.com.

Multiple factors likely contribute to T-cell exclusion, but cancer-associated fibroblasts (CAFs), specifically TGFβ-activated myofibroblastic CAFs, are increasingly recognized as major orchestrators. Via secretion of extracellular matrix proteins and other immunomodulators, these CAFs create fibrotic, immunosuppressive TMEs that restrict T-cell migration into tumor nests (7). The respective mechanisms underlying ICI resistance for the three immune subtypes and the corresponding methods for reversing are illustrated in **Figure 1**.

This review synthesizes recent advances in our understanding on TGFβ-activated CAF-mediated T-cell exclusion, evaluates challenges in targeting TGFβ/CAFs, and highlights downstream effectors (e.g., LRRC15, NOX4) as precision therapeutic targets to restore ICB sensitivity.

## 2 T-cell exclusion: prognostic and predictive implications

As the main activators of antitumor immunity, tumor infiltrating T cells have been intensely investigated as prognostic and predictive biomarkers. However, until now, they have experienced limited clinical application because of modest efficacy and lack of applicable evaluation methods. The effect of spatial distribution of tumor infiltrating T cells on patients’ prognosis has been recognized for more than two decades (8, 9). The concept of T-cell exclusion, however, has emerged with the advent of the immunotherapy era, despite two decades of research on T-cell spatial distribution. A triple-negative breast cancer study revealed 10-year survival rates of 80% for inflamed tumors, 60% for excluded tumors, and 40% for desert tumors. Spatial phenotypes were prognostic independent of factors like nodal status, tumor size, and age (10). Artificial intelligence (AI) was recently leveraged to analyze non-small cell lung cancer tumors revealing 44% inflamed tumors, 37% excluded tumors, and 19% desert tumors. This could provide a supplementary biomarker to immunohistochemistry (11).

Besides its role in prognosis, T-cell exclusion is evolving as a potential biomarker for immunotherapy resistance. In two cohorts of patients with advanced NSCLC treated with monotherapy ICBs, the overall response rate to ICBs in patients with immune-excluded phenotype was 11.5%, compared with 26.8% and 11.2% in patients with inflamed and immune-desert phenotypes, respectively. Median progression-free survival and overall survival among patients with the immune-excluded phenotype were also significantly shorter than those with the inflamed phenotype (2.2 and 14.0 months vs. 4.1 and 24.8 months) (11). T-cell exclusion signatures could also predict resistance to ICBs (12), while a T cell-inflamed signature (13, 14) or a T-cell infiltration signature (15) corresponds highly with a clinical response to ICBs in multiple tumor types. T-cell exclusion might be responsible for both primary (16) as well as acquired ICB resistance (17).

## 3 MyoCAFs: architects of T-cell exclusion

CAFs are one of the most abundant and critical components of the TME, which not only provide physical support but also play a key role in metabolic and immune reprogramming of the TME (18). Evidence shows that CAFs dominate the complex cell–cell interactions in the TME (19). The role of CAFs in promoting the establishment of an immunosuppressive TME in various cancers is increasingly appreciated. Highly stromagenic cancers, such as pancreatic cancer, respond particularly poorly to ICB treatments. Lung fibrosis impairs T cell-mediated tumor control and limits the benefit of ICB treatment in NSCLC (20).

As the main drivers of stromagenesis and fibrosis, CAFs directly orchestrate CD8+ T-cell exclusion (21), thereby shaping an immunologically cold TME. This causal relationship is evidenced by dose-dependent correlations between CAF density and the excluded tumor phenotype (22). However, CAFs within tumors are not uniform, but have extensive heterogeneity, with at least two major CAFs subtypes, myoCAFs and inflammatory CAFs (iCAFs) (23–25). MyoCAFs have a matrix-producing myofibroblastic phenotype with high expression of α-smooth muscle actin (αSMA). By contrast, iCAFs have a secretory phenotype with the ability to generate immunomodulatory molecules such as interleukin 6 and C-X-C motif chemokine ligand 12 (26). Determining the heterogeneity within the CAFs is indispensable to better understand and treat cancers.

By using multicolor flow cytometry, a seminal breast cancer study identified four CAF subsets with distinct immunomodulatory capacities, with the αSMA-high CAF-S1 subset most deeply involved in establishing an immunosuppressive TME (27). Their subsequent work based on single-cell RNA sequencing (scRNA-seq) uncovered delineated further heterogeneity within the CAF-S1 subset revealing the role of myofibroblastic CAF subsets (ecm/TGFβ/wound-myoCAF) in orchestrating T-cell exclusion and ICB resistance (28). This role of the myoCAFs has been corroborated by another group (29).

MyoCAFs are increasingly recognized as key mediators in driving immune exclusion and resistance to ICB therapies through dual mechanisms as follows: (1) promoting fibrotic TMEs and (2) forming functional alliances with immunosuppressive immune cells, particularly tumor-associated macrophages (TAMs). Spatial analysis from a landmark Chinese study revealed that myoCAFs exhibit intensive cross-talk with TAMs to create physical (ECM and collagen organization) and biochemical (chemotaxis regulation) barriers that restrict cytotoxic T-lymphocyte penetration (30). The tumor-promoting role of myoCAFs has been validated through pan-cancer analysis encompassing 18 malignancies using a cell-type deconvolutional approach. The results showed that cancers with high numbers of myoCAFs have poorer prognosis and lower drug response sensitivity (31), while targeting myoCAFs increases infiltration of cytotoxic CD8+ T cells into the tumor parenchyma and improves ICB efficacy (32). These collective findings position myoCAFs as central architects of the immune-excluded phenotype and critical facilitators of tumor progression.

## 4 TGFβ-activated CAFs: fueling ICB resistance and T-cell exclusion

TGFβ is a well-established inducer of myoCAFs, in contrast to interleukin 1 and fibroblast growth factor, which drive the formation of iCAFs (33–35). Studies have highlighted the crucial role of TGFβ-activated CAFs in predicting resistance to ICBs across various cancers.

A Canadian study found that TGFβ-dysregulated CAF ECM genes predict PD1/PDL1 resistance better than established biomarkers like the tumor mutation burden, cytolytic activity, T cell-inflamed signature, TGFβ expression alone, and the CAF signature (36). Another study showed that a stroma/epithelial-to-mesenchymal transition (EMT)/TGFβ signature negatively associates with pembrolizumab response across cancers (37). TGFβ-responsive CAF signatures define poor prognosis subtypes and predict ICB resistance in colorectal cancer (38), including microsatellite instability high/mismatch repair-deficient cancers normally susceptible to ICBs (39). For urothelial carcinoma, TGFβ signaling in CAFs associated with ICB resistance in both metastatic (7) and neoadjuvant settings (40). TGFβ-activated CAFs are also responsible for T-cell exclusion in ovarian cancer (41).

Besides the significant link between TGFβ-activated CAFs and ICB resistance, *in vivo* and *in vitro* models recapitulating the role TGFβ-activated CAFs in driving T-cell exclusion have been developed. The EMT6 (epithelial cell line) and MC38 mouse mammary carcinoma models seem to naturally exhibit a T cell-excluded phenotype. Therapeutic blockade of PDL1 or TGFβ alone achieved little or no effect in these mice, while blocking both PD-L1 and TGFβ exhibited a significant reduction in tumor burden by promoting significant infiltration of T cells into the tumor nest (7).

A more elegant work was reported by a Spanish team, who generated mouse strains bearing combined mutations of *Apc*, *Kras*, *Tgfbr2*, and *Trp53* in intestinal stem cells. These mice developed highly invasive colon cancer with 90% prevalence and reproduced key features of the T-cell exclusion phenotype induced by a prominent TGFβ-activated stroma. PD1/PDL1 inhibitors provoked a limited response in this model. In contrast, inhibition of TGFβ unleashed a potent response to PD1/PDL1 inhibitors (42). However, that study did not explain why these autochthonous tumors developed a TGFβ-activated stroma. Recently, another group performed unbiased analysis for regulators of the TME using spatial functional genomics and found that the *Tgfbr2* knockout in cancer cells could increase TGFβ bioavailability, drive TGFβ-mediated CAF activation, and convert the TME to a fibro-mucinous state inducing T-cell exclusion and ICB resistance (43). This conclusion echoes the findings from the Spanish team and might provide a reproducible method to build mouse models of T cell-excluded tumors by knockout of the *Tgfbr2* gene.

Another team described a preclinical model named the skin tumor array by microporation (STAMP), to characterize spatiotemporal immune response patterns, and showed enriched myofibroblasts and TGFβ activation in excluded tumors. Interestingly, immune phenotypes seem stochastic, not predetermined by genetics. Fibroblasts have a complex dual role—they drive exclusion but also orchestrate inflammation by recruiting T cells. Depleting fibroblasts increased desert tumors in STAMP revealing the multifaceted functions of fibroblasts in shaping immune phenotypes (16). These findings underscore the need to elucidate specific mechanisms and fibroblast heterogeneity.

**Figure 2:**
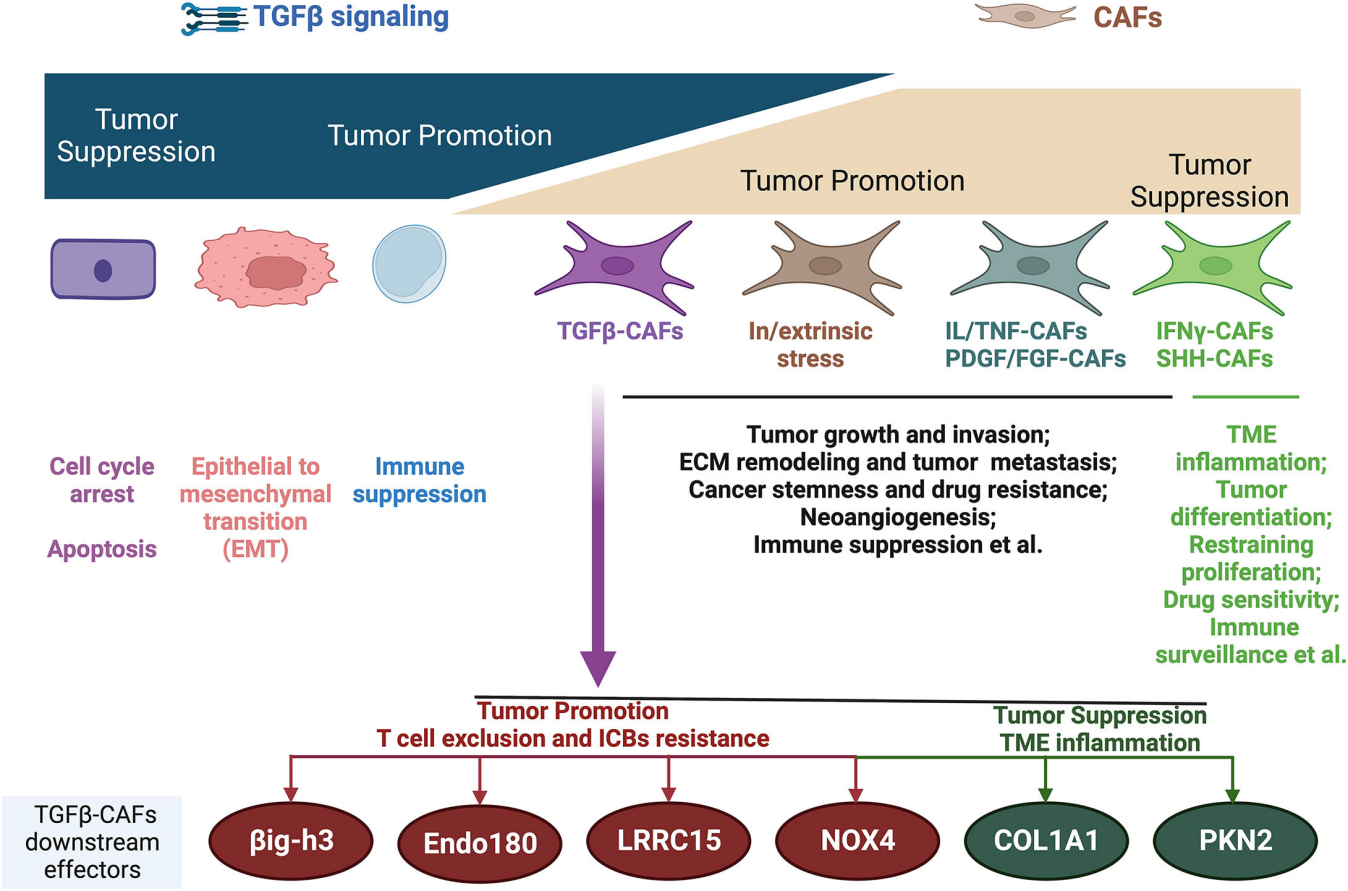
Biphasic functions of TGFβ and CAFs during cancer progression and the main mechanisms. TGFβ-CAFs emerge as the main contributors to T-cell exclusion immune phenotype and ICB resistance, with several downstream molecules being leveraged to reverse T-cell exclusion and ICB resistance. However, it should be noted that the downstream effectors of TGFβ-CAFs are functionally multifaceted, with some downstream molecules having tumor-suppressive roles. IL, interleukin; TNF, tumor necrosis factor; PDGF, platelet-derived growth factor; FGF, fibroblast growth factor; IFNγ, interferon-γ; SHH, Sonic Hedgehog; ECM, extracellular matrix; TME, tumor microenvironment. Image created with Biorender.com.

Despite established links between TGFβ-activated CAFs and PD-1/PD-L1 resistance, therapeutic targeting remains fraught with paradoxes. Non-specific ablation of myoCAFs (e.g., αSMA+ depletion or Sonic hedgehog inhibition) not only fails to restore immune infiltration but accelerates tumor progression (44–47) likely due to inadvertent disruption of tumor-restraining CAF subsets within the population (**Figure 2**) (48, 49).

Similarly, while preclinical models demonstrate synergy between TGFβ inhibitors and ICBs (7, 42), clinical trials reveal limited efficacy and unforeseen risks—TGFβ blockade may paradoxically promote metastasis and on-target toxicity (50–52). These contradictions stem from TGFβ’s context-dependent duality: it constrains early tumors via growth suppression yet drives advanced disease through stromal activation and immune evasion (52). TGFβ’s dual tumor-suppressive/promoting roles demand subset-specific targeting strategies.

## 5 Precision targeting TGFβ-activated CAFs

Studies suggested that nonspecific targeting of CAFs or TGFβ signaling in tumors might not suppress tumor progression; therefore, specific targeting of TGFβ-activated CAFs subtypes might be needed to improve the clinical outcome. Determining the molecular drivers of TGFβ-activated CAFs in T-cell exclusion might provide the understanding needed to design more effective immunotherapeutic approaches and address the unmet clinical need in ICB-resistant T cell-excluded cancers.

The well-established downstream effectors of TGFβ-activated CAFs are the ECM proteins, the dysregulation of which predicts a failure of PD-1 blockade across cancers (36, 53). One study identified ECM protein collagen type XIII alpha 1 chain as a biomarker for the TGFβ-responsive CAF subpopulation that produces chemokines to limit recruitment of dendritic cells and T cells into the tumor nest, thus leading to poor responsiveness to anti-PD1/PDL1 therapy in lung cancer (20). However, they did not provide ways to specifically target these distinct CAFs. βig-h3 (also known as induced TGFβ) is another ECM protein that is regulated by TGFβ in CAFs. βig-h3-secreting CAFs within the stroma can sequester T cells in the stroma leading to a T cell-excluded phenotype. Depletion of βig-h3 could drive the accumulation of CD8+ T cells and enhance the antitumoral response (54). Anti-βig-h3 antibody has exhibited promising anti-tumor efficacy by reshaping TMEs (55). Therefore, βig-h3 represents a novel and promising immunological target among ECM proteins in cancer.

A seminal work from Genentech reported an unbiased assessment of fibroblast heterogeneity in pancreatic ductal adenocarcinoma tissues by bulk and scRNA-seq of stromal cells. They identified LRRC15 as a marker of TGFβ-driven myoCAFs, which were the dominant fibroblasts in advanced tumors across multiple human cancer types (56). LRRC15 is a transmembrane protein that is physiologically involved in cell–cell and cell–ECM interactions (57). The LRRC15+ CAF signature has been found to not only predicts worse survival but also poor treatment response in patients receiving anti-PDL1 therapy (56, 58, 59). Recently, LRRC15 serves as a definitive marker for a terminally differentiated myoCAF subset that orchestrates immune-excluded TMEs (60). Further work revealed that LRRC15+ CAF depletion leads to persistent reduction in the tumor burden and significantly boosts anti-PD1/PDL1 treatment responses (61). However, targeting LRRC15 clinically seems difficult due to its poorly characterized functions and signaling pathways. Interestingly, recent advances in antibody–drug conjugates (ADCs) have produced a wide range of “magic bullets” to address previously unmet medical needs (62). LRRC15 shows highly restricted expression in some pro-tumorigenic and immunosuppressive CAFs; therefore, it has become a good cancer-specific antigen for therapeutic targeting. LRRC15-targeted ADCs, such as the ABBV-085 ADC conjugated with monomethyl auristatin E (MMAE) payloads, are under development, and promising results are emerging (57, 63).

Endocytic receptor 180 (also known as urokinase-type plasminogen activator receptor-associated protein, encoded by the *MRC2* gene, is an endocytic receptor participating in collagenolysis and ECM turnover. The expression of Endo180 is physiologically restricted to normal tissue fibroblasts. Recent advances revealed that Endo180 is upregulated on a subset of myoCAFs and plays a critical role in mediating TGFβ-induced collagen accumulation and contractility. Genetic deletion of *ENDO180* profoundly limited tumor growth and metastasis (64). The same groups’ subsequent work revealed the role of Endo180+ CAFs in driving the T-cell exclusion phenotype. High levels of Endo180 in tumors predict a poor response to PD1/PDL1 inhibitor therapy, and knockout of *ENDO180* in CAFs promotes T-cell infiltration and enhanced sensitivity to PD1/PDL1 inhibitor therapy (22). The fact that Endo180 is specifically upregulated on a subset of tumor-promoting myoCAFs and is a constitutively recycled transmembrane receptor makes Endo180 an ideal target antigen to design an ADC. Currently, an Endo180-ADC with a payload of MMAE has been tested in a preclinical sarcoma tumor xenograft model resulting not only in tumor regression but also in a significant reduction of metastasis. Endo180-ADC could be rapidly internalized into target cells and cause specific cell death in Endo180-expressing CAFs (65).

NOX4 has been identified as another major downstream target of TGFβ-activated CAFs to promote T-cell exclusion and anti-PD1/PDL1 resistance (66). NOX4 catalyzes the reduction of O_2_ to produce reactive oxygen species (ROS), which are required for TGFβ-dependent myofibroblast transdifferentiation in various organ fibroses and malignancies (67–69). Pharmacological inhibition of NOX4 using GKT137831, an oral drug developed as an anti-fibrotic agent, abrogated TGFβ-dependent ROS production, myofibroblast transdifferentiation, and slow tumor growth (70). By using CAF-rich murine tumor models with a T-cell exclusion phenotype, the role of NOX4 inhibition in normalizing myoCAFs to a quiescent phenotype and overcoming T-cell exclusion by increasing intratumoral T-cell infiltration has been revealed. By contrast, TGFβ inhibition alone could prevent, but not reverse, CAF differentiation. This preclinical study showed that NOX4 inhibition could restore the response to immunotherapy by overcoming CAF-mediated T-cell exclusion (66).

Recent findings reveal tumor-restraining functions of some TGFβ-activated CAF effectors emphasizing the need for a nuanced understanding. Collagen 1A1, a TGFβ-induced effector, suppresses tumors when deleted in myoCAFs by recruiting immunosuppressive myeloid-derived suppressor cells (71, 72). Preserving collagen while targeting other CAF mediators is suggested. Similarly, the TGFβ mediator, Rho effector kinase protein kinase N2 (PKN2), restrains invasion, as its deletion switches myoCAFs to inflammatory CAFs (73). These results highlight the heterogeneity of CAFs, as well as myoCAFs, cautioning against blanket CAF targeting and warranting elucidating the multifaceted TGFβ–CAF roles.

## 6 Discussion

TGFβ-activated CAFs have emerged as critical drivers of immunotherapy resistance and the T cell-excluded phenotype across various cancers. However, blanket targeting of CAFs or TGFβ has yielded mixed results clinically. Therefore, further research should focus on identifying and selectively targeting the specific downstream mediators induced by TGFβ in the subpopulations of tumor-promoting CAFs. Promising targets in early investigation include ECM proteins like βig-h3, membrane receptors like Endo180 and LRRC15, and cytosolic enzymes like NOX4. These effectors promote the immunosuppressive and T cell-excluding effects of TGFβ-activated CAFs and are absent or minimal in normal tissues. Drugs targeting them may provide more precise immunotherapies. Particularly promising are ADC drugs targeting specific membrane proteins induced by TGFβ in pro-tumorigenic CAFs exemplified by LRRC15 and Endo180-targeted ADCs.

Additionally, single-cell profiling has revealed further heterogeneity within CAF populations, so future work should examine how different CAF subtypes interact to shape distinct immune phenotypes. A nuanced understanding of CAF subsets may enable selective elimination of pro-tumorigenic CAFs while preserving anti-tumorigenic subtypes. Defining the spatiotemporal dynamics of CAF activation and crosstalk with other stromal and immune components is also warranted. Overall, research on the multi-faceted roles of CAFs and their subpopulations will uncover new strategies to overcome resistance and expand the benefits of cancer immunotherapy to more patients.

## Glossary

ADCs: antibody–drug conjugates
AI: artificial intelligence
BTLA: molecule B and T-lymphocyte attenuator
CAFs: cancer-associated fibroblasts
ECM: extracellular matrix
EMT: epithelial-to-mesenchymal transition
Endo180: endocytic receptor 180
iCAFs: inflammatory CAFs
ICBs: immune checkpoint blockers
LRRC15: leucine-rich repeat containing 15
MMAE: monomethyl auristatin E
myoCAFs: myofibroblastic CAFs
NOX4: NADPH oxidase 4
NSCLC: non-small-cell lung cancer
OX40: TNF superfamily member 4
OX40L: ligand of OX40
PD1: programmed cell death 1
PDL1: programmed death ligand 1
PKN2: Rho effector kinase protein kinase N2
ROS: reactive oxygen species
scRNA-seq: single-cell RNA sequencing
STAMP: skin tumor array by microporation
TAMs: tumor-associated macrophages
TGFβ: transforming growth factor beta
TME: tumor microenvironment
αSMA: α-smooth muscle actin
βig-h3: induced TGFβ.
